# Inhibition of H3K27me3 Histone Demethylase Activity Prevents the Proliferative Regeneration of Zebrafish Lateral Line Neuromasts

**DOI:** 10.3389/fnmol.2017.00051

**Published:** 2017-03-13

**Authors:** Beier Bao, Yingzi He, Dongmei Tang, Wenyan Li, Huawei Li

**Affiliations:** ^1^State Key Laboratory of Medical Neurobiology, Medical College of Fudan UniversityShanghai, China; ^2^ENT Institute and Otorhinolaryngology Department of Affiliated Eye and ENT Hospital, State Key Laboratory of Medical Neurobiology, Fudan UniversityShanghai, China; ^3^Key Laboratory of Hearing Medicine of National Health and Family Planning CommissionShanghai, China; ^4^Institutes of Biomedical Science, Fudan UniversityShanghai, China; ^5^The Institutes of Brain Science and the Collaborative Innovation Center for Brain Science, Fudan UniversityShanghai, China

**Keywords:** GSK-J4, H3K27 demethylation, hair cell, regeneration, zebrafish

## Abstract

The H3K27 demethylases are involved in a variety of biological processes, including cell differentiation, proliferation, and cell death by regulating transcriptional activity. However, the function of H3K27 demethylation in the field of hearing research is poorly understood. Here, we investigated the role of H3K27me3 histone demethylase activity in hair cell regeneration using an *in vivo* animal model. Our data showed that pharmacologic inhibition of H3K27 demethylase activity with the specific small-molecule inhibitor GSK-J4 decreased the number of regenerated hair cells in response to neomycin damage. Furthermore, inhibition of H3K27me3 histone demethylase activity dramatically suppressed cell proliferation and activated caspase-3 levels in the regenerating neuromasts of the zebrafish lateral line. GSK-J4 administration also increased the expression of *p21* and *p27* in neuromast cells and inhibited the ERK signaling pathway. Collectively, our findings indicate that H3K27me3 demethylation is a key epigenetic regulator in the process of hair cell regeneration in zebrafish and suggest that H3K27me3 histone demethylase activity might be a novel therapeutic target for the treatment of hearing loss.

## Introduction

Death of sensory hair cells is the major cause of hearing impairment. In the mature mammalian inner ear, the majority of damaged hair cells do not regenerate, and this leads to irreversible and permanent hearing loss (Forge et al., 1993; Warchol et al., 1993; Brigande and Heller, 2009). In contrast, non-mammalian vertebrates are capable of regenerating lost sensory hair cells after damage (Balak et al., 1990; Lombarte et al., 1993; Harris et al., 2003; Pisano et al., 2014). New hair cells are frequently produced by proliferation of non-sensory supporting cells, which are the source of hair cell precursors that subsequently differentiate into new hair cells and supporting cells (Raphael, 1992; Stone and Cotanche, 1994; Jones and Corwin, 1996). Discovering and understanding key pathways and mediators in non-mammalian vertebrates during the process of proliferative regeneration will likely provide new treatments for hearing restoration in mammals.

The zebrafish lateral line consists of neuromasts that are distributed along the head and body surface, and each neuromast contains a group of hair cells that are similar to mammalian inner ear sensory hair cells in terms of both morphology and function (Raible and Kruse, 2000; Whitfield, 2002; Nicolson, 2005). In addition, zebrafish lateral line hair cells can rapidly regenerate after damage and almost all hair cell recovery occurs within 72 h, and these characteristics make the zebrafish lateral line an excellent model to study hair cell regeneration (Williams and Holder, 2000; Harris et al., 2003; López-Schier and Hudspeth, 2006; Hernández et al., 2007; Ma et al., 2008). Further, the superficial location of the lateral line hair cells on the surface of the body makes them ideal for experimental manipulation and *in vivo* imaging.

Although much work has been performed on transcription factors and signaling pathways over the years (Ma et al., 2008; Lin et al., 2013; Jacques et al., 2014; Jiang et al., 2014; Romero-Carvajal et al., 2015), the epigenetic mechanisms such as histone modification that govern hair cell regeneration are still largely unknown (He et al., 2014, 2016a; Tang et al., 2016). Methylation of basic amino acid residues in histone proteins is a crucial epigenetic modification for the regulation of gene expression. Recent evidence suggests that tri-methylation of histone H3 at lysine 27 (H3K27me3) is associated with gene silencing, whereas demethylation of H3K27 by specific demethylases correlates with transcriptional activation (Cao et al., 2002; Müller et al., 2002; Agger et al., 2007; Lan et al., 2007; Zhou et al., 2011). The KDM6 family Utx (also known as Kdm6a) and Jmjd3 (also known as Kdm6b) proteins are typical histone H3K27 demethylases that have been shown to be critical for the regulation of biological processes by opening up compact chromatin and making it accessible to transcription factors (Agger et al., 2007; Ramadoss et al., 2012; Jiang et al., 2013; Kartikasari et al., 2013).

Previous studies have demonstrated that *Utx*/*Jmjd3*-null mice die at birth, which reveals an important role for the KDM6 family of demethylases in the regulation of gene expression programs during the determination of cell fate (Shpargel et al., 2012, 2014; Welstead et al., 2012). For example, it was reported that loss of function of *Jmjd3* in the developing retina reduces transcription factor *Bhlhb4* expression and causes protein kinase C-positive bipolar cell subsets to fail to differentiate, implying that the *Jmjd3* demethylase is associated with the development of sensory organs (Iida et al., 2014). Recently, the importance of H3K27me3 demethylase in the regeneration process has been highlighted in several papers (Stewart et al., 2009; Faralli et al., 2016). Faralli et al. (2016) demonstrated that *Utx m*ediates muscle gene expression through demethylation of H3K27me3, and deficiency in *Utx* results in defective muscle repairin satellite cell-mediated muscle regeneration. In addition, the *Jmjd3* gene lies in the blastema in regenerating fins of zebrafish larvae, and it is strongly upregulated during regeneration, while *Jmjd3* knockdown larvae exhibit a pronounced inability to regenerate caudal fins (Stewart et al., 2009). Together, these findings have revealed a physiological role for active H3K27 demethylation *in vivo*. However, the role of H3K27 demethylase activity in hair cell regeneration remains to be established. Therefore, our objective was to determine whether H3K27 demethylation has a functional link to hair cell regeneration.

In this study, we used the zebrafish lateral line system to investigate the effects of H3K27me3 histone demethylases on hair cell regeneration, as well as the underlying mechanisms for such effects. We observed reduced regenerated hair cells in the H3K27me3 demethylation-inhibited larvae during the regeneration process compared to control larvae after neomycin damage. We showed that inhibition of H3K27me3 demethylase not only suppressed the proliferation of supporting cells, but it also induced apoptosis in the regenerating neuromasts of the zebrafish lateral line. We also found that GSK-J4 might inhibit cell cycle progression via the ERK signaling molecule and the tumor suppressors p21 and p27. These results extend our knowledge of epigenetic regulators in hair cell regeneration and suggest that the H3K27 demethylase might serve as a potential therapeutic target in human hearing loss.

## Materials and methods

### Zebrafish embryos and drug administration

All zebrafish animal experiments were performed following the institutional guidelines approved by the Institutional Animal Care and Use Committee of Fudan University, Shanghai. GSK-J4 (Selleck) was dissolved in dimethyl sulfoxide (DMSO, Sigma-Aldrich) at a stock concentration of 10 mM and further diluted to the desired concentrations in fresh egg water. DMSO was selected as the vehicle control group. GSK-J1, inactive control compound GSK-J2, and the MAPK/ERK kinase inhibitor U0126 were purchased from MedChem Express. For hair cell damage, neomycin sulfate (Sigma-Aldrich) was added to 5 days post-fertilization (dpf) larvae at a final concentration of 400 μM and incubated for 1 h. This was followed by three rinses in fresh egg water, and the zebrafish larvae were allowed to recover for 24 h or 48 h at 28.5°C. Drug solutions were replaced after 24 h. The larval zebrafish were then fixed with 4% paraformaldehyde (PFA) in phosphate buffered saline (PBS) at 4°C until further processing.

### Cell proliferation and analysis

Proliferating cells in neuromasts were labeled by adding 10 mM 5-bromo-2-deoxyuridine (BrdU; Sigma-Aldrich) to the fresh egg water for 24 or 48 h at 28.5°C. Larvae were then fixed with 4% PFA overnight at 4°C, and BrdU incorporation was detected by fluorescent immunostaining. The fixed larvae were washed three times in PBS containing 0.5% Triton X-100 (PBT-2) and placed in 2 N HCl for 0.5 h at 37°C. Larvae were blocked in 10% normal goat serum for 1 h at room temperature and incubated with the monoclonal anti-BrdU primary antibody overnight at 4°C. The next day, the larvae were washed three times for 10 min each with PBT-2 and then incubated with the secondary antibody for 1 h at 37°C. Fluorescently labeled larvae were imaged with a Leica confocal fluorescence microscope (TCS SP8; Leica, Wetzlar, Germany).

### Immunohistochemistry

Larvae were fixed with 4% PFA and were permeabilized with PBT-2 for 30 min followed by incubation in blocking solution for 1 h. Primary antibodies were then added overnight at 4°C with rocking. The following antibodies were used as primary antibodies:anti-GFP (1:1,000 dilution; Abcam, Cambridge, UK), anti-Sox2 (1:500 dilution; Abcam), anti-myosin VI (1:500 dilution), anti-cleaved caspase-3 (1:500 dilution; Cell Signaling Technology Inc., Danvers, MA, USA), and anti-H3K27me3 (1:1,000 dilution; Abcam). After three washes of 20 min, Alexa Fluor 488–, 594–, and/or 647–conjugated secondary antibodies (Jackson ImmunoResearch Laboratories, West Grove, PA, USA) were added at a 1:500 dilution in blocking solution and incubated overnight at 4°C with rocking. Nuclei were labeled with 4,6-diamidino-2-phenylindole (DAPI; 1:800 dilution; Invitrogen, Carlsbad, CA, USA) for 20 min at room temperature. For image collection, Z-sections were taken at 1 μm intervals through the depth of the neuromast. For analyses, maximum intensity projections were generated, and images were processed using Photoshop software (Adobe). Cell counts were performed at the time of imaging by viewing the images using a Nikon Eclipse Ni Fluorescence Microscope (Nikon Instruments) using a 40 × objective. Double-labeled cells in neuromasts were counted on a confocal microscope using a 63× objective (TCSSP8; Leica, Wetzlar, Germany). BrdU+ cells having a shape identical to that of a hair cell or supporting cell and corresponding to the exact location of a neuromast were counted.

### FM1-43FX labeling

The vital dye FM1-43FX (Invitrogen)—which enters mature hair cells through mechanotransduction-dependent activity—was applied at a concentration of 3 μM to live 5 dpf larvae for 45s in the dark. After quickly rinsing three times with fresh water, the larvae were anesthetized in 0.02% MS-222 (Sigma-Aldrich) and fixed with 4% PFA in PBS for 2 h at room temperature or overnight at 4°C.

### Western blot analysis

Total proteins were isolated from whole larvae at 5 dpf using RIPA buffer supplemented with *complete* EDTA-free Protease Inhibitor Cocktail (Roche, Mannheim, Germany). Total proteins were separated on SDS-polyacrylamide gels and transferred onto PVDF membranes (Immobilon-P; Millipore, Bedford, MA, USA). The membranes were blocked with 5% non-fat dried milk in TBST (20 mM Tris-HCl (pH 7.5), 500 mM NaCl, and 0.1% Tween-20) for 1 h at room temperature and then blotted overnight with primary antibodies at 4°C. After washing with TBST, the membranes were blotted with horseradish peroxidase-conjugated secondary antibody (Jackson ImmunoResearch Laboratories, Inc.) for 1 h at room temperature. The reactions were detected using ECL Prime Western Blotting Detection Reagent (GE Healthcare, Wauwatosa, WI, USA). The following antibodies were used as primary antibodies: anti-cleaved caspase-3 (1:500 dilution; Cell Signaling Technology Inc.), anti-H3K27me3 (1:1,000 dilution; Abcam), anti-ERK1/2 (1:1,000 dilution; Abcam), and anti-phosphorylated ERK1/2 (p-ERK1/2) (1:1,000 dilution; Abcam). All figures showing quantitative analysis include data from at least three independent experiments. Quantitative analysis of the western blot data was carried out using Photoshop software (Adobe).

### Whole-mount *in situ* hybridization

The probes used in the *in situ* hybridization were amplified by PCR from zebrafish embryo cDNA using the following primers and cloned into the pGEM-T Easy Vector (Promega). *p21* forward: 5′ -acaagcggatcctacgttca-3′; *p21* reverse: 5′-ctacgagacgaatgcagctc-3′; *p27* forward: 5′- acttcgacttttccacgcac-3′; *p27* reverse: 5′-tgctttattgttgagtgccaga-3′. Digoxigenin-labeled antisense RNA probes were generated by *in vitro* transcription using T7 or SP6 RNA polymerase (Promega). Regular whole-mount *in situ* hybridization of zebrafish embryos was performed as previously described (Thisse and Thisse, 2008). Briefly, the embryos were depigmented with 1-phenyl-2-thiourea (Sigma-Aldrich), euthanized in MS-222, and fixed overnight with 4% PFA at 4°C. The fixed embryos were washed in PBS with 0.1% Tween-20 (PBST) and placed in 100% methanol at −20°C for dehydration. Prior to use, they were rehydrated in a graded methanol series and washed three times for 5 min with PBST. To permeabilize the embryos, proteinase K (10 μg/mL in PBST) was added for 50 min and the embryos were refixed in 4% PFA for 20 min. After washing in PBST, the embryos were prehybridized at 65°C for ≥2 h in hybridization buffer. For hybridization, the labeled probes were added to the hybridization buffer at 65°C overnight. After washing for 15 min with 75, 50, and 25% hybridization buffer and 2 × SSCT (20 × SSC, Life technologies; 0.1% Tween-20) and for 30 min twice in 0.2 × SSC at 65°C, the embryos were blocked for at least 2 h at 4°C in blocking buffer (Roche) and were incubated with preabsorbed sheep anti-digoxigenin-AP Fab fragments (Roche) at a 1:4,000 dilution in blocking buffer overnight at 4°C. The next day, the embryos were washed 4 × 30 min with 2 mg/mL BSA in PBST and 3 × 5 min in staining buffer (100 mM Tris (pH 9.5), 100 mM NaCl, and 0.1% Tween-20). Afterwards, the embryos were stained with BM purple AP substrate (Roche) in the dark. Finally, the color reaction was stopped by adding PBST, and the embryos were observed under a bright field microscope (Nikon Instruments). Sites of binding were identified as blue-black dots.

### Statistical analysis

Cells in the first two lateral line neuromasts were counted. All data are presented as the mean ± SEM. Data were analyzed using SigmaPlot (version 12.0 for Windows; Systat Software Inc., CA, USA). Comparisons between two groups and multiple groups were analyzed using an unpaired *t*-test (2-tail) or one-way ANOVA (see figure legends for details). A *p* < 0.05 was considered statistically significant.

## Results

### GSK-J4 treatment impairs hair cell regeneration in the zebrafish lateral line

**Figure 1:**
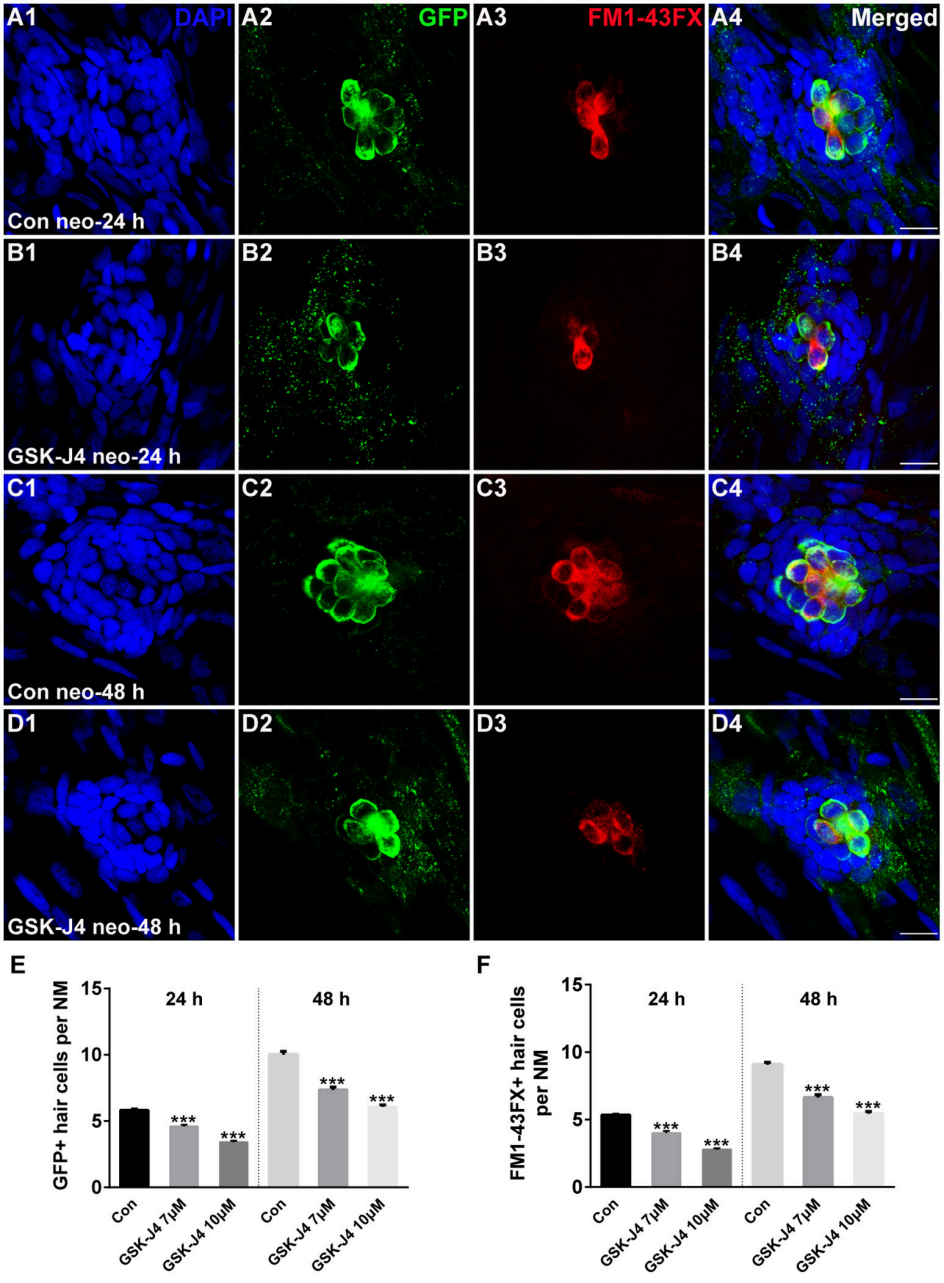
**GSK-J4 impaired hair cell regeneration after neomycin damage. (A–D)** GSK-J4 reduced the numbers of GFP-positive (green) and FM1-43FX-positive (red) hair cells compared with DMSO-treated controls. Scale bars = 10 μm. **(E,F)** Quantitative analysis of the number of GFP-positive **(E)** or FM1-43FX-positive **(F)** hair cells per neuromast (NM) at different time points in DMSO-treated control and GSK-J4-treated larvae. In the 24-h group, *n* = 40 neuromasts (20 larvae) per group; in the 48-h group, *n* = 28 neuromasts (14 larvae) per group. ^***^p < 0.0001. Bars are mean ± sem. [24-h group: One-way ANOVA; GFP-positive cells: *F*_(2, 117)_ = 96.94; FM1-43FX-positive cells: *F*_(2, 117)_ = 114. 48-h group: One-way ANOVA; GFP-positive cells: *F*_(2, 81)_ = 88.96; FM1-43FX-positive cells: *F*_(2, 81)_ = 93.85].

GSK-J4 is an ethyl ester derivative of the H3K27 demethylase inhibitor GSK-J1, and it has been used in various studies of the function of H3K27me3 histone demethylases (Kruidenier et al., 2012; Liu et al., 2015; Hofstetter et al., 2016; Xie et al., 2016). To study the role of GSK-J4 in hair cell regeneration, we treated 5 dpf zebrafish larvae with 400 μM neomycin for 1 h to kill mature lateral line hair cells (Supplemental Figure 1) and then placed the larvae in 6-well plates with 7 or 10 μM GSK-J4 for 24 or 48 h. Fish expressing a membrane-bound GFP under the control of the *brn3c* promoter (*brn3c*:gfp) (Xiao et al., 2005) were used to label hair cells in the lateral line. At 24 h post-treatment (hpt), hair cells of DMSO-treated control and GSK-J4-treated larvae had no observable morphological differences; however, control neuromasts had an average of 5.8 ± 0.13 GFP-positive hair cells (Figures 1A1,2; *n* = 40 neuromasts), while the larvae treated with GSK-J4 had fewer hair cells in neuromasts (Figures 1B1,2;E; 7 μM GSK-J4: 4.6 ± 0.12 GFP-positive hair cells; 10 μM GSK-J4: 3.4 ± 0.12 GFP-positive hair cells; *n* = 40 neuromasts per treatment group; *p* < 0.0001). At 48 hpt, there were still apparent differences in the number of GFP-positive hair cells between DMSO-treated control larvae and GSK-J4-treated larvae (Figures 1C1,2;D1,2;E; DMSO control: 10 ± 0.26 GFP-positive hair cells; 7 μM GSK-J4: 7.4 ± 0.21 GFP-positive hair cells; 10 μM GSK-J4: 6.1 ± 0.14 GFP-positive hair cells; *n* = 28 neuromasts per group; *p* < 0.0001).

To determine whether the hair cells in GSK-J4-treated neuromasts contained functional mechanotransduction channels, live larvae were transferred to the well of a 6-well plate containing the vital dye FM1-43FX for 45 s before fixation. FM1-43FX enters into hair cells through open mechanotransduction channels (Meyers et al., 2003). Our quantification indicated that GSK-J4-treated neuromasts had significantly fewer FM1-43FX-positive cells as compared to those generated from DMSO-treated larvae at both 24 and 48 h (Figures 1A3–D3; A4–D4; F; *p* < 0.0001), suggesting that normal neuromast hair cell regeneration is significantly disrupted by treatment with GSK-J4.

### GSK-J4 treatment results in reduced proliferation

**Figure 2:**
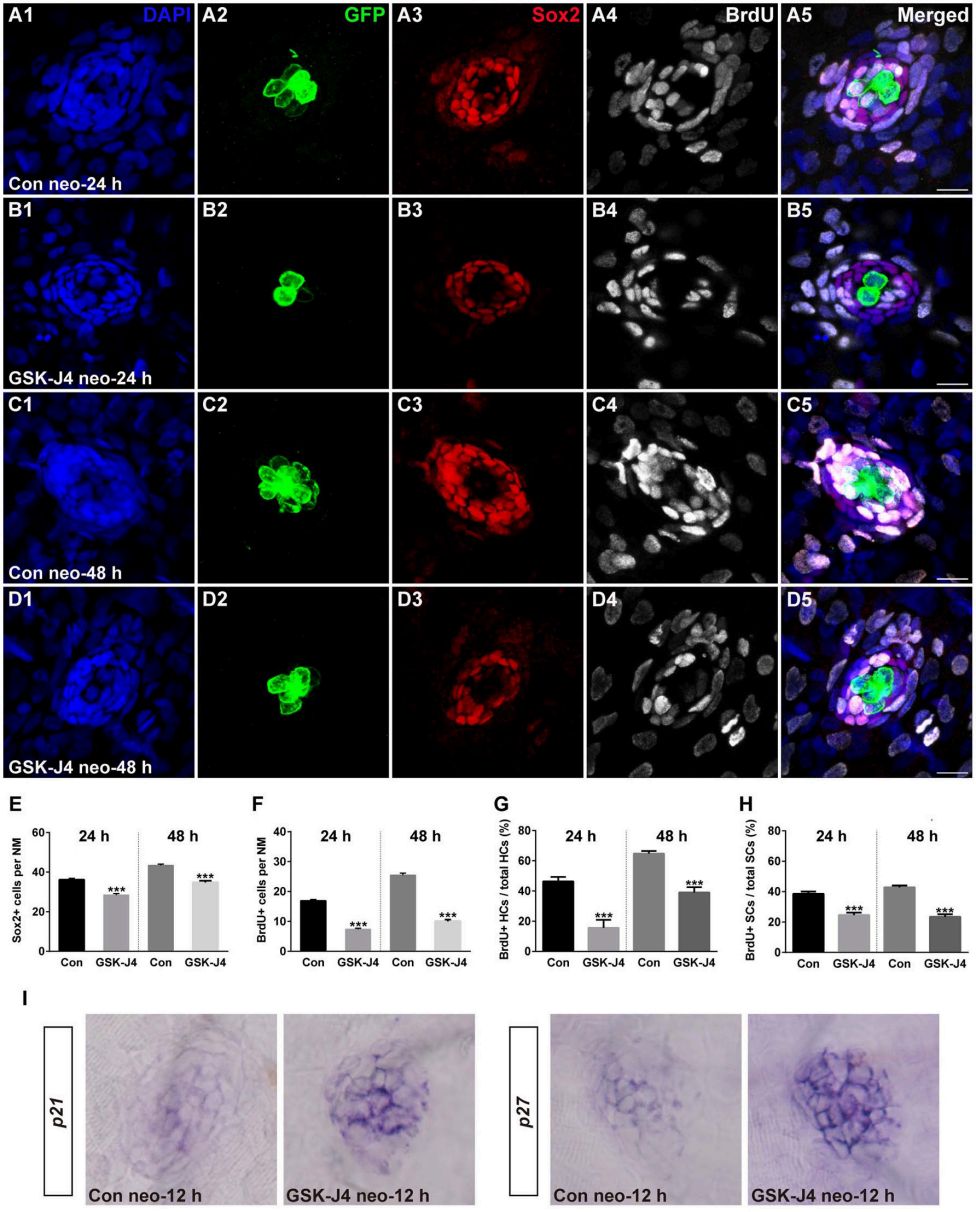
**GSK-J4 reduced proliferation in regenerating neuromast cells. (A–D)** 5 dpf larvae were treated with 400 μM neomycin for 1 h followed by GSK-J4 exposure for 24 or 48 h in the presence of BrdU. GSK-J4 significantly reduced the numbers of Sox2-positive (red) and BrdU-positive (white) replicating cells. Scale bars = 10 μm. **(E,F)** Quantification of Sox2-positive and BrdU-positive cells per neuromast (NM) in DMSO-treated control larvae and 10 μM GSK-J4-treated larvae at 24 or 48 h following neomycin damage. In the 24-h group, *n* = 30 neuromasts of DMSO-treated control larvae (15 larvae) and *n* = 24 neuromasts of 10 μM GSK-J4-treated larvae (12 larvae); in the 48-h group, *n* = 36 neuromastsof DMSO-treated control larvae (18 larvae) and *n* = 24 neuromasts of 10 μM GSK-J4-treated larvae (12 larvae). ^***^p < 0.0001. (24-h group: Sox2-positive cells: unpaired *t*-test, two-tailed, *t* = 7.412, *df* = 52; BrdU-positive cells: unpaired *t*-test, two-tailed, *t* = 13.86, *df* = 52. 48-h group: Sox2-positive cells: unpaired *t*-test, two-tailed, *t* = 7.463, *df* = 58; BrdU-positive cells: unpaired *t*-test, two-tailed, *t* = 15.6, *df* = 58). Bars are mean ± sem. **(G,H)** Quantitative analysis of the proportion of BrdU-positive hair cells **(G)** or BrdU-positive supporting cells **(H)** in control and GSK-J4-treated larvae at 24 or 48 h after neomycin damage. In the 24-h group, *n* = 30 neuromasts of DMSO-treated control larvae (15 larvae) and *n* = 24 neuromasts of 10 μM GSK-J4-treated larvae (12 larvae); in the 48-h group, *n* = 36 neuromasts of DMSO-treated control larvae (18 larvae) and *n* = 24 neuromasts of 10 μM GSK-J4-treated larvae (12 larvae). ^***^p < 0.0001. (24-h group: BrdU-positive HCs: unpaired *t*-test, two-tailed, *t* = 5.309, *df* = 52; BrdU-positive SCs: unpaired *t*-test, two-tailed, *t* = 6.294, *df* = 52. 48-h group: BrdU-positive HCs: unpaired *t*-test, two-tailed, *t* = 7.279, *df* = 58; BrdU-positive SCs: unpaired *t*-test, two-tailed, *t* = 9.546, *df* = 58). Bars are mean ± sem. **(I)** Localization of the *p21* and *p27* genes by whole-mount *in situ* hybridization in GSK-J4-treated and DMSO-treated control larvae. GSK-J4 treatment significantly increased the expression of *p21* and *p27* in regenerating neuromasts at 12 hpt. (*n* = 20–26 neuromasts per group). Results from single representative neuromasts are shown.

We investigated the effect of GSK-J4 on the supporting cells after neomycin-induced damage. Immunostaining for Sox2, a marker of supporting cells (Hernández et al., 2007), indicated that the numbers of supporting cells per neuromast were significantly decreased compared with DMSO-treated control groups at 24 or 48 h after 10 μM GSK-J4 exposure (Figures 2A3–D3;E; *p* < 0.0001). These results suggest that H3K27me3 histone demethylase activity is involved in the production of both hair cells and supporting cells in the regenerating neuromast.

The decrease in regenerated hair cells might be due to the impairment of cell proliferation and the death of newly generated hair cells. To help determine which of these possibilities is occurring, we labeled S-phase cells in the lateral line neuromasts throughout the regeneration period following neomycin-induced hair cell loss. After neomycin damage, 5 dpf zebrafish larvae were incubated in fresh egg water containing 10 mM BrdU with GSK-J4 (10 μM) for 24 or 48 h. At 24 hpt, cell proliferation, as indicated by the number of BrdU-positive cells per neuromast, was significantly impaired after GSK-J4 treatment compared with DMSO-treated control larvae (Figures 2A4;B4;F; 24 hpt DMSO control: 16.8 ± 0.51 BrdU-positive cells per neuromast, *n* = 30; GSK-J4-treated: 7.3 ± 0.43 BrdU-positive cells per neuromast; *n* = 24, *p* < 0.0001), and similar results were seen at 48 hpt with DMSO-treated controls averaging 25.4 ± 0.71 BrdU-positive cells per neuromast and GSK-J4-treated larvae averaging 10 ± 0.58 BrdU-positive cells per neuromast (Figures 2C4;D4;F; *p* < 0.0001).

Furthermore, to distinguish the new mitotically regenerated hair cells, we double-labeled the zebrafish larvae with anti-BrdU and anti-GFP antibodies at 24 or 48 h after neomycin damage. Few GFP-positive/BrdU-positive hair cells were observed in GSK-J4-treated groups at either of the time points evaluated. At 24 hpt, the proportion of BrdU and GFP double-labeled cells to the total number of GFP-positive hair cells in DMSO-treated control neuromasts was 46.4 ± 2.96% (Figures 2A1,2,5; *n* = 30 neuromasts), whereas the proportion in 10 μM GSK-J4-treated larvae was significantly lower at 15.6 ± 5.32% (Figures 2B1,2,5; *n* = 24 neuromasts) (Figure 2G; *p* < 0.0001). After 48 h of continuous BrdU incorporation, GSK-J4-treated larvae still had significantly fewer BrdU and GFP double-positive hair cells per neuromast when compared with DMSO-treated control neuromasts (Figures 2C1,2,5; D1,2,5; G; DMSO control: 64.7 ± 1.74%, *n* = 36 neuromasts; 10 μM GSK-J4: 39 ± 3.47%, *n* = 24 neuromasts; *p* < 0.0001). The ratio of BrdU and Sox2 double-labeled cells to the total number of Sox2-positive supporting cells in the GSK-J4-treated neuromasts were also drastically reduced at both 24 and 48 h (Figure 2H; *p* < 0.0001). Taken together, these results show that H3K27me3 histone demethylase activity is required for cell proliferation in the regenerating neuromast.

To further demonstrate a role for H3K27 demethylation in hair cell regeneration, zebrafish larvae were treated with GSK-J1, a previously identified H3K27me3-specific demethylase inhibitor (Kruidenier et al., 2012), and hair cells were labeled with another typical hair cell marker, myosin VI. As a control for these experiments, we used GSK-J2, an inactive isomer of GSK-J1 that does not have any specific activity, making it an appropriate negative control for studies involving GSK-J1 (Kruidenier et al., 2012). Our results showed that GSK-J1 treatment significantly decreased the replaced hair cells (Supplemental Figure 2) in the neuromast of larvae when compared to the control group treated with GSK-J2 (*p* < 0.0001) (Supplemental Figure 2). Similarly, the cell proliferation was also reduced by GSK-J1 treatment when compared to control (GSK-J2) larvae (*p* < 0.0001) (Supplemental Figure 2).

We continuously incubated zebrafish larvae in BrdU in the presence of GSK-J4 without neomycin treatment. Larvae were then immunostained for both myosinVI and BrdU. In DMSO-treated control larvae not exposed to neomycin, a low level of BrdU-positive cells was observed at the examined time point (Supplemental Figure 3), and this is consistent with both the slow addition of hair cells due to the growth of the neuromasts and with normal turnover of hair cells. No significant differences in proliferative cell and hair cell numbers were seen in the presence of GSK-J4 compared with the DMSO-treated control for both BrdU-positive (*p* > 0.1) and myosinVI-positive (*p* > 0.1) cells (Supplemental Figure 3), suggesting that H3K27me3 demethylase activity was activated to induce cell proliferation during the hair cell regeneration process.

Previous studies have demonstrated that the cyclin-dependent kinase (CDK) inhibitors p21 and p27 are required for regulating the cell cycle programs (Becker and Bonni, 2004; Singhal et al., 2005; Wang et al., 2009; Yoon et al., 2012; Marqués-Torrejón et al., 2013). To further understand whether the reduced proliferation in regenerating neuromasts was due to alteration in the expression of these CDK inhibitors, we analyzed their mRNA expression by *in situ* hybridization. Our results revealed that GSK-J4 treatment increased the expression of *p21* and *p27* mRNA in the regenerating neuromast cells (Figure 2I). Thus, we hypothesized that H3K27me3 demethylases promote cell proliferation via the suppression of *p21* and *p27* expression.

### GSK-J4 induces apoptosis in neuromast cells

**Figure 3:**
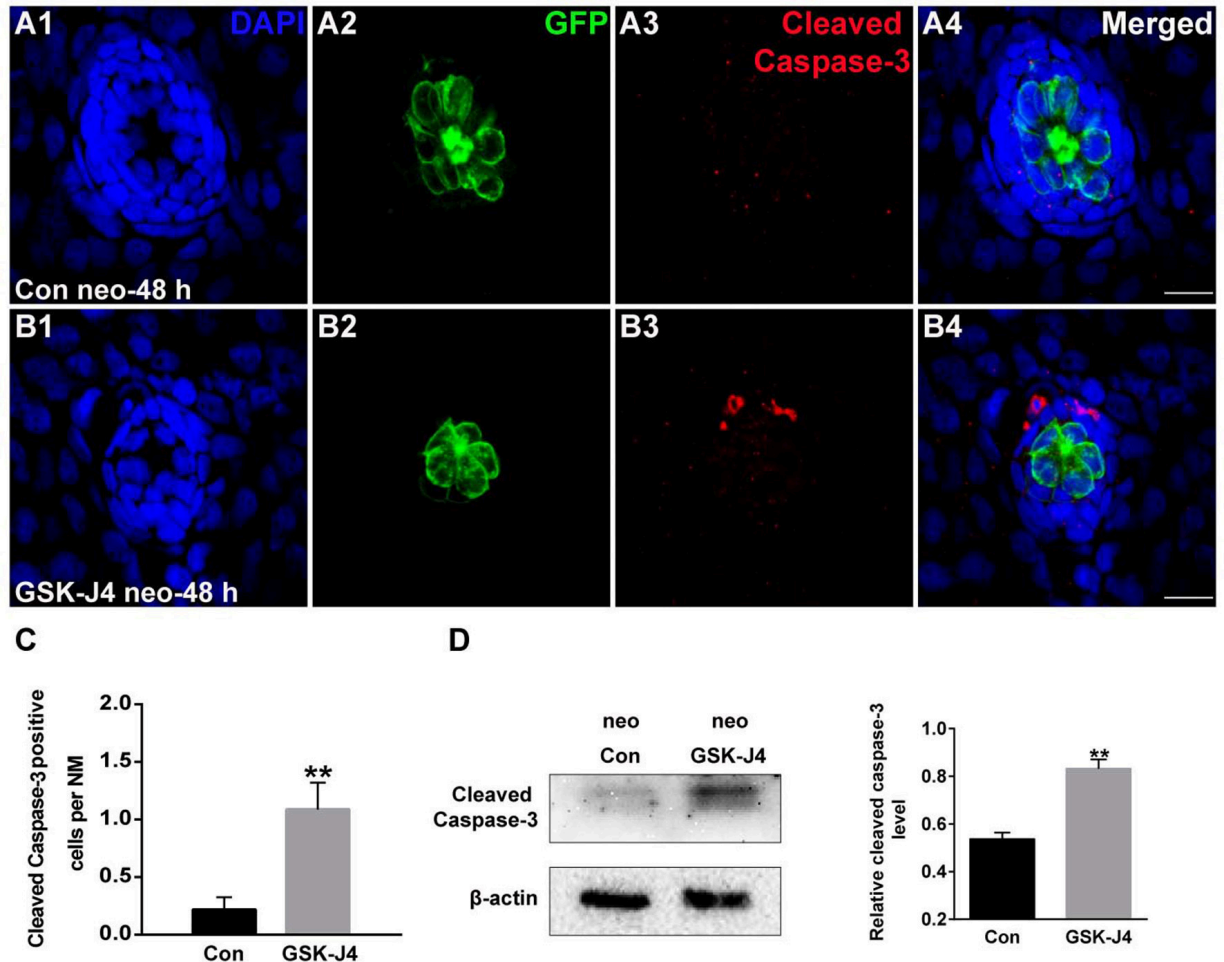
**GSK-J4 induced apoptosis in the regenerating neuromasts. (A,B)** Cleaved caspase-3 staining in the neuromasts from a DMSO-treated control larva **(A)** and a GSK-J4-treated larva **(B)** at 48 h after neomycin damage. Scale bar = 10 μm. **(C)** Quantitative analysis of the number of cleaved caspase-3-labeled cells in DMSO-treated control and GSK-J4-treated larvae. *n* = 24 neuromasts (12 larvae) per group. ^**^p < 0.001 (unpaired *t*-test, two-tailed, *t* = 3.368, *df* = 44, *p* = 0.0016). **(D)** Cleaved-caspase-3-specific western blot analysis of whole larvae treated with either DMSO (neo Con; *n* = 6 larvae) or 10 μM GSK-J4 (neo GSK-J4; *n* = 6 larvae) for 48 h. The relative expressions of cleaved-caspase-3/β-actin were calculated. ^**^p < 0.001.

To evaluate the effect of GSK-J4 on apoptosis, we labeled larvae with an anti-cleaved caspase-3 antibody. As shown in Figure 3A, we occasionally detected cleaved caspase-3-positive cells in DMSO-treated control larvae. However, the emergence of cleaved caspase-3-positive cells was significantly greater in larvae treated with 10 μM GSK-J4 for 48 h (Supplemental Figure 4; Figures 3B,C; *p* < 0.001). This was confirmed by the western blot analysis of proteins from zebrafish larvae (Figure 3D). Taken together, these results suggest that GSK-J4 induces apoptosis in the lateral line neuromast.

**Figure 4:**
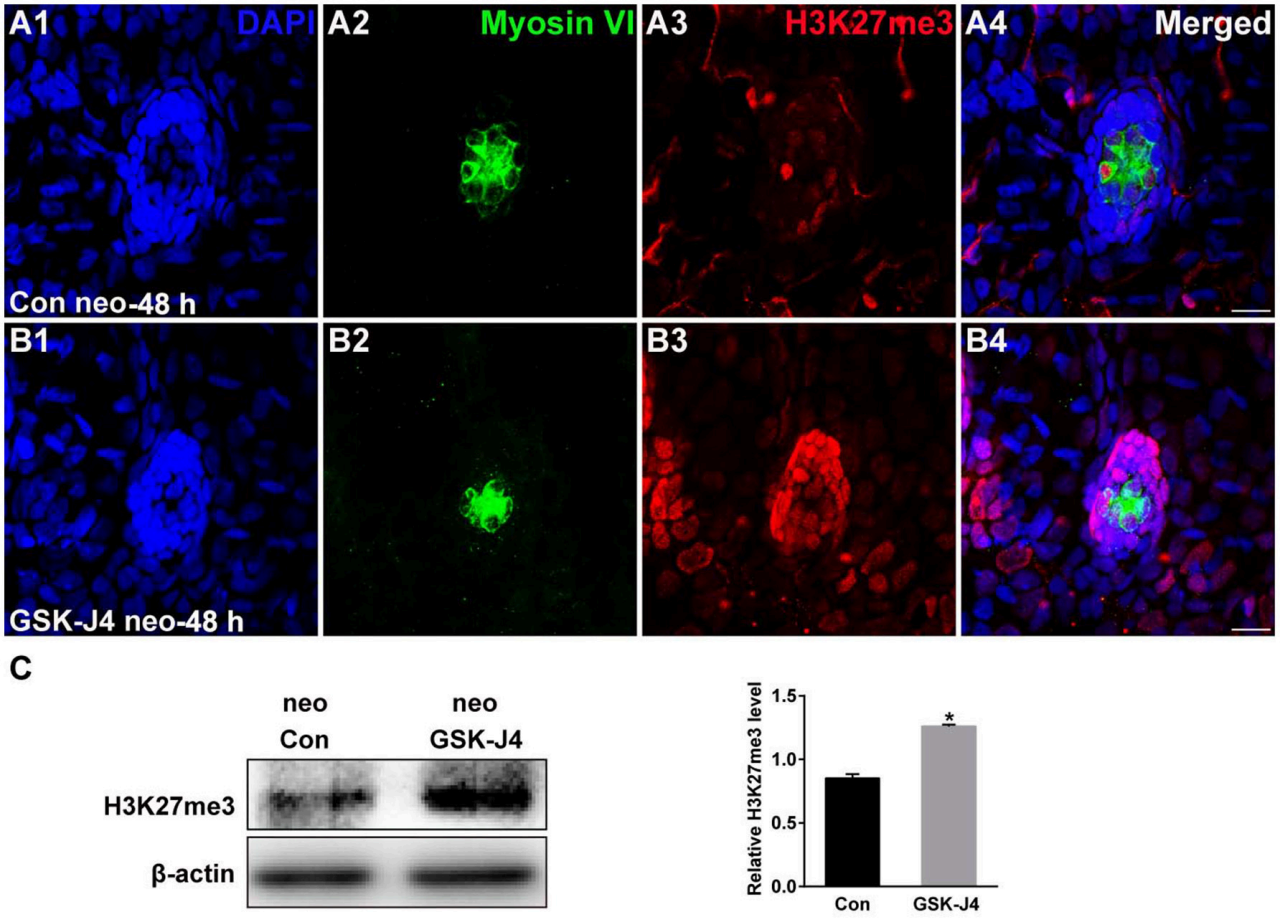
**GSK-J4 increased the levels of H3K27me3. (A,B)** Immunohistochemistry results showing H3K27me3 expression in neuromasts of DMSO-treated control (Con) larvae (*n* = 5 larvae) and of 10 μM GSK-J4-treated larvae (*n* = 6 larvae) for 48 h. Scale bar = 10 μm. **(C)** Western blot analysis was performed to evaluate the expressions of H3K27me3 and β-actin in whole larvae that were treated with either DMSO (neo Con; *n* = 6 larvae) or GSK-J4 (neo GSK-J4; *n* = 6 larvae) for 48 h. ^*^p < 0.05.

The status of H3K27 trimethylation is a direct marker of KDM6 activity. Therefore, to confirm that H3K27 demethylases were inhibited by GSK-J4 under our experimental conditions, we examined the global level of H3K27me3 in the zebrafish larvae. Immunofluorescence and western blot analysis demonstrated that compared to the DMSO-treated control, the H3K27me3 level was significantly increased in zebrafish following GSK-J4 treatment (Figure 4). Taken together, the above results demonstrate that GSK-J4 effectively inhibits cell proliferation and induces cell death in neuromasts most likely through inhibition of H3K27 demethylases.

### GSK-J4 treatment decreases phosphorylation of ERK in zebrafish larvae

**Figure 5:**
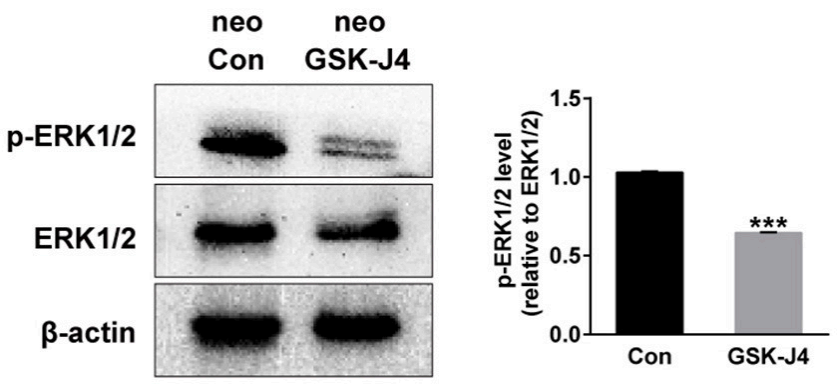
**GSK-J4 decreased phosphorylation of ERK1/2 (p-ERK1/2) in zebrafish larvae**. Western blot analysis was performed to detect the expressions of p-ERK1/2 and ERK1/2 in DMSO-treated control larvae (neo Con; *n* = 6 larvae) and GSK-J4-treated larvae (neo GSK-J4; *n* = 6 larvae). ^***^p < 0.0001.

Because ERK is mainly associated with proliferative stimuli, and because the MAPK/ERK pathway is tightly related to cell cycle control (Torii et al., 2006; Meloche and Pouysségur, 2007), we investigated whether this pathway is relevant to the hair cell regeneration defect caused by GSK-J4 treatment. First, we examined the levels of ERK1/2 and phospho-ERK1/2, an active form of ERK, in the control and GSK-J4-treated zebrafish by western blotting. As shown in Figure 5, the phosphorylation levels of ERK1/2 at the end of the 48 h period of GSK-J4treatment were significantly decreased when compared with controls, suggesting that ERK is part of the mechanism triggered by GSK-J4 to induce the hair cell regeneration defect. We next investigated the mechanisms by which ERK might influence hair cell regeneration by analyzing cell cycle-related genes. We treated embryos with ERK inhibitor U0126 (20 μM) for 12 h and found that the mRNA levels of *p21* and *p27* were increased after ERK inhibition when compared to the control (Supplemental Figure 5), suggesting that a decrease in p-ERK is involved in the alteration of *p21* and *p27* mRNA expression during hair cell regeneration.

## Discussion

Histone-modifying enzymes play important roles in most biological systems, but their contributions in hearing, for example, in hair cell regeneration, remain largely unknown. In a recent study, we reported that histone demethylase LSD1 favors hair cell regeneration in the zebrafish lateral line by targeting the Wnt/β-catenin and Fgf signaling pathways (He et al., 2016a). However, whether other histone methyltransferases and/or demethylases are involved in hair cell regeneration is unknown. H3K27me3 is a key epigenetic modification that has various functional roles in cellular processes such as cell differentiation, inflammation, tumorigenesis, and cellular reprogramming by targeting distinct transcription factors (Hong et al., 2007; Sen et al., 2008; Estarás et al., 2012; Kruidenier et al., 2012; Ramadoss et al., 2012; Welstead et al., 2012; Ntziachristos et al., 2014; Salminen et al., 2014). In this study, using a selective JMJD/UTX-specific enzymatic inhibitor, we reveal that inhibition of the H3K27 demethylase activity inhibits hair cell regeneration following neomycin damage.

Because hair cell replacement can be produced from proliferative progenitors in the larval zebrafish lateral line neuromast, new hair cells are generated primarily by proliferation of supporting cells that then differentiate into hair cells (Harris et al., 2003; López-Schier and Hudspeth, 2006; Ma et al., 2008). Here, S-phase cells were analyzed by BrdU incorporation, and supporting cells were labeled using Sox2 immunocytochemistry. We observed that BrdU-labeled cells in the larval zebrafish lateral line were generally located in the periphery of the neuromast following neomycin exposure, and we are currently developing a Sox2/BrdU double-labeling protocol to accurately describe hair cell regeneration and the progenitors in this model system. Our results are similar to the previous observations of lateral line hair cell regeneration, confirming the hypothesis that supporting cells are the hair cell precursors in the neuromast of zebrafish lateral line and that they proliferate after damage stimuli to renew both hair cells and supporting cells (Ma et al., 2008). Further, inhibition of H3K27me3 histone demethylase activity with GSK-J4 dramatically decreased the numbers of proliferative cells and regenerated hair cells, which supports the idea that this is a potentially important regulator of supporting cell proliferative regeneration. However, we cannot exclude that ongoing proliferation is occurring in the neuromasts during our experiments, and Harris et al. (2003) have provided direct evidence that there is ongoing proliferation in neuromasts of normal, undamaged animals. Our data indicated that GSK-J4 treatment has no effect on hair cell number in the absence of neomycin damage, suggesting that H3K27 histone demethylase activity is initiated during the regeneration process to help ensure that the correct number of hair cells is regenerated.

A possible underlying cause for the decreased numbers of hair cells in regenerating neuromasts upon H3K27 demethylase inhibition might be epigenetic regulation of the cell cycle. p21 and p27 are important CDK inhibitors that regulate cell cycle arrest in a variety of cell types (Becker and Bonni, 2004; Singhal et al., 2005; Wang et al., 2009; Yoon et al., 2012; Marqués-Torrejón et al., 2013). In this study, to understand the regulation of these CDK inhibitors, we investigated their mRNA expression and found that *p21* and *p27* were significantly up-regulated following inhibition of H3K27me3 histone demethylase activity by GSK-J4 administration, suggesting that H3K27me3 demethylases might control hair cell regeneration by regulating *p21* and *p27*expression. These findings are supported by previous reports (Qiu et al., 2015; Hofstetter et al., 2016). Hofstetter et al. (2016) showed that abrogation of H3K27 demethylase activity by the small molecular inhibitor GSK-J4 or by KDM6 genetic deficiency leads to cell cycle arrest and cell death in differentiating ESCs. Next, in the global transcriptome analyses of GSK-J4-treated differentiating ESCs, they confirmed that the expression of p21 and H3K27 demethylase activity were negatively correlated. Further investigation is needed to test whether GSK-J4 regulates *p21* and *p27* expression in a direct way or not. We could not completely exclude the possibility that GSK-J4 could regulate a set of other cell-proliferation and/or cell death-associated genes. It would be helpful to develop a better understanding of the epigenetic regulation of hair cell regeneration by RNA-sequencing and whole genome ChIP-seq analyses.

Next we asked whether the cell cycle arrest in the presence of GSK-J4 is paralleled by an increase in apoptosis. We therefore performed cell death analysis by cleaved caspase-3 immunofluorescence staining. Our data clearly showed that GSK-J4 increased the number of cleaved caspase-3-positive cells in neuromasts compared to controls, confirming that inhibition of H3K27me3 demethylase leads to cell death in neuromasts during regeneration mainly through caspase-3 activation. In addition, we performed western blot analyses with total protein isolated from whole larvae that were treated with GSK-J4 or DMSO, suggesting that the cleaved caspase-3 levels of GSK-J4-treated zebrafish larvae were significantly higher than those of DMSO-treated controls. We could not exclude the possibility that proteins might be affected in other tissues where H3K27me3 demethylases are expressed because the proteins used for western blot analysis were isolated from the whole larvae, not isolated neuromasts.

Many transcription factors and signaling pathways are involved in the regulation of hair cell regeneration (Ma et al., 2008; Jacques et al., 2014; Jiang et al., 2014; Wu et al., 2014; Romero-Carvajal et al., 2015; Rubbini et al., 2015; He et al., 2016b), so we then investigated which underlying mechanisms are associated with hair cell regeneration and GSK-J4 treatment following neomycin damage. Our data showed that inhibition of H3K27me3 histone demethylases by GSK-J4 significantly down-regulated the phosphorylation of ERK1/2 in zebrafish. ERK1/2 are members of the family of mitogen-activated protein kinases (MAPKs), which regulate a multitude of cellular processes such as cell proliferation, differentiation, migration, and survival (Widmann et al., 1999; Torii et al., 2006; Meloche and Pouysségur, 2007). For example, it has been reported that inhibition of the ERK1/2 signaling pathway in pancreatic cancer cell lines leads to a cessation of cell proliferation accompanied by cell cycle arrest (Gysin et al., 2005). Previous data reported that the basal level of ERK activation in normal hair cells is important for their survival, and inhibition of ERK1/2 in cochlear explants leads to significant hair cell loss *in vitro* (Battaglia et al., 2003). *Erk2* knockout mice have significantly fewer hair cells after noise exposure, indicating that activation of ERK2 in hair cells plays an important protective role against noise-induced hearing loss (Kurioka et al., 2015). Because ERK1/2 is activated by the upstream protein kinase MEK, MEK inhibitors such as U0126 have been frequently used to analyze the function of ERK1/2 signaling in a wide array of biological processes. Previous work in our laboratory demonstrated that attenuated phosphorylation of pRb through inhibition of the MEK/ERK pathway by U0126 decreased the proliferation of supporting cells and resulted in decreased expression of CDKs in the damaged neonatal chicken utricle, suggesting that the ERK signaling pathway is involved in hair cell regeneration (Wu et al., 2014). In our study, we found that the ERK1/2 pathway is part of the mechanism triggered by H3K27me3 inhibition to impair hair cell regeneration in zebrafish lateral line neuromasts. Further analyses will show whether the ERK1/2 pathway interferes with cell cycle control in this condition. We observed that inhibition of ERK activation by U0126 increased the expression of *p21* and *p27* mRNA levels, suggesting that a decrease in p-ERK seems to be involved in the alteration of *p21* and *p27* mRNA expression during hair cell regeneration. These results are consistent with previous data showing that ERK activity contributes to the down-regulation of p21 and p27 that precedes cell cycle progression (Kortylewski et al., 2001; Villanueva et al., 2007; Hwang et al., 2009; Zhu et al., 2016; Seo et al., 2017). The possibility that other signaling pathways are involved in controlling *p21* and *p27* during hair cell regeneration cannot be excluded from our results. Further analyses will be needed to investigate the detailed relationship among H3K27me3 histone demethylases, the expression of *p21* and *p27*, and ERK activity as well as to identify other genes regulated by H3K27me3 demethylase through microarray analysis or RNA-sequencing following H3K27me3 demethylase knockdown.

In summary, we observed that inhibition of H3K27me3 histone demethylase activity results in decreased cell proliferation, inactivation of ERK signaling, and increased cell death in zebrafish during hair cell regeneration. Although the zebrafish lateral line is a powerful model system for studying hair cell regeneration, there are differences between zebrafish and mammals. Unlike the mammalian inner ear, there is no compartmentalization of fluid spaces in the zebrafish lateral line, and the stereocilia of these lateral line hair cells extend into the surrounding water. There is also no separation into inner and outer hair cells within a neuromast. Furthermore, unlike their mammalian counterparts, zebrafish have the remarkable ability to regenerate hair cells after hair cell death (López-Schier and Hudspeth, 2006; Ma et al., 2008; Pisano et al., 2014). Follow-up mammalian studies are required to elucidate whether a similar role of H3K27me3 histone demethylase activity is observed in the mammalian inner ear and what the underlying mechanisms are. Our study not only uncovers a novel insight into the epigenetic regulatory mechanisms of hair cell regeneration, but it also provides an attractive approach for the treatment of hearing loss.

## Author contributions

BB and HL conceived and designed the work. BB and YH wrote the manuscript. BB and YH performed the zebrafish experiments. DT, WL, and HL performed data analyses. All authors discussed the data, and all authors reviewed the manuscript.

### Conflict of interest statement

The authors declare that the research was conducted in the absence of any commercial or financial relationships that could be construed as a potential conflict of interest.

## Supplementary material

Supplementary Figure 1 **Five dpf zebrafish larvae treated with 400 μM neomycin to kill mature lateral line hair cells**.

Supplementary Figure 2 **GSK-J1 impaired zebrafish hair cell regeneration. (A–F)** 5 dpf larvae were treated with 400 μM neomycin for 1 h followed by GSK-J1 exposure for 24 or 48 h in the presence of BrdU. GSK-J1 significantly reduced the numbers of myosinVI-positive (green) hair cells and BrdU-positive (red) replicating cells. Scale bars = 10 μm. **(G,H)** Quantification of myosinVI-positive and BrdU-positive cells per neuromast (NM) in DMSO-treated control larvae (Con), 15 μM GSK-J2-treated control larvae, and 15 μM GSK-J1-treated larvae at 24 or 48 h following neomycin damage. In the 24-h group, *n* = 28 neuromasts of DMSO-treated control larvae (14 larvae), *n* = 30 neuromasts of 15 μM GSK-J2-treated control larvae (15 larvae), and *n* = 20 neuromasts of 15 μM GSK-J1-treated larvae (10 larvae); in the 48-h group, *n* = 18 neuromasts of DMSO-treated control larvae (9 larvae), *n* = 22 neuromasts of GSK-J2-treated control larvae (11 larvae), and *n* = 18 neuromasts of 15 μM GSK-J1-treated larvae (9 larvae). ^***^*p* < 0.0001. Bars are mean ± sem.

Supplementary Figure 3 **GSK-J4 incubation did not affect the pattern of cell proliferation during development. (A,B)** In the larvae not exposed to neomycin, supporting cell proliferation was at a low level overall for both DMSO-treated control and GSK-J4-treated larvae. **(C,D)** Quantification of myosinVI-positive hair cells and BrdU-positive cells per neuromast (NM) in DMSO-treated 5 dpf control larvae (Con) and 10 μM GSK-J4-treated 5 dpf larvae for 24 h. *n* = 20 neuromasts of DMSO vehicle control larvae (10 larvae) and *n* = 28 neuromasts of 10 μM GSK-J4-treated larvae (14 larvae).

Supplementary Figure 4 **Effects of GSK-J4 on apoptosis in the zebrafish body**. Detection of cell apoptosis by cleaved caspase-3 staining in the body of zebrafish larvae exposed to DMSO (Con) or 10 μM GSK-J4 at 48 h following neomycin damage. The cleaved caspase-3-positive cells are indicated by white arrows, and the neuromasts are outlined.

Supplementary Figure 5 **Effects of ERK1/2 inhibition on the expression of *p21* and *p27***. The mRNA levels of *p21* and *p27* in regenerating neuromasts were increased after U0126 treatment at 12 hpt when compared to the respective control larvae (*n* = 16–20 neuromasts per group).
