# Supplementary figures and images for: Inhibition of H3K27me3 Histone Demethylase Activity Prevents the Proliferative Regeneration of Zebrafish Lateral Line Neuromasts
Source: Front Mol Neurosci. 2017 Mar 13;10:51. doi: 10.3389/fnmol.2017.00051 (PMC5346882; doi:10.3389/fnmol.2017.00051)

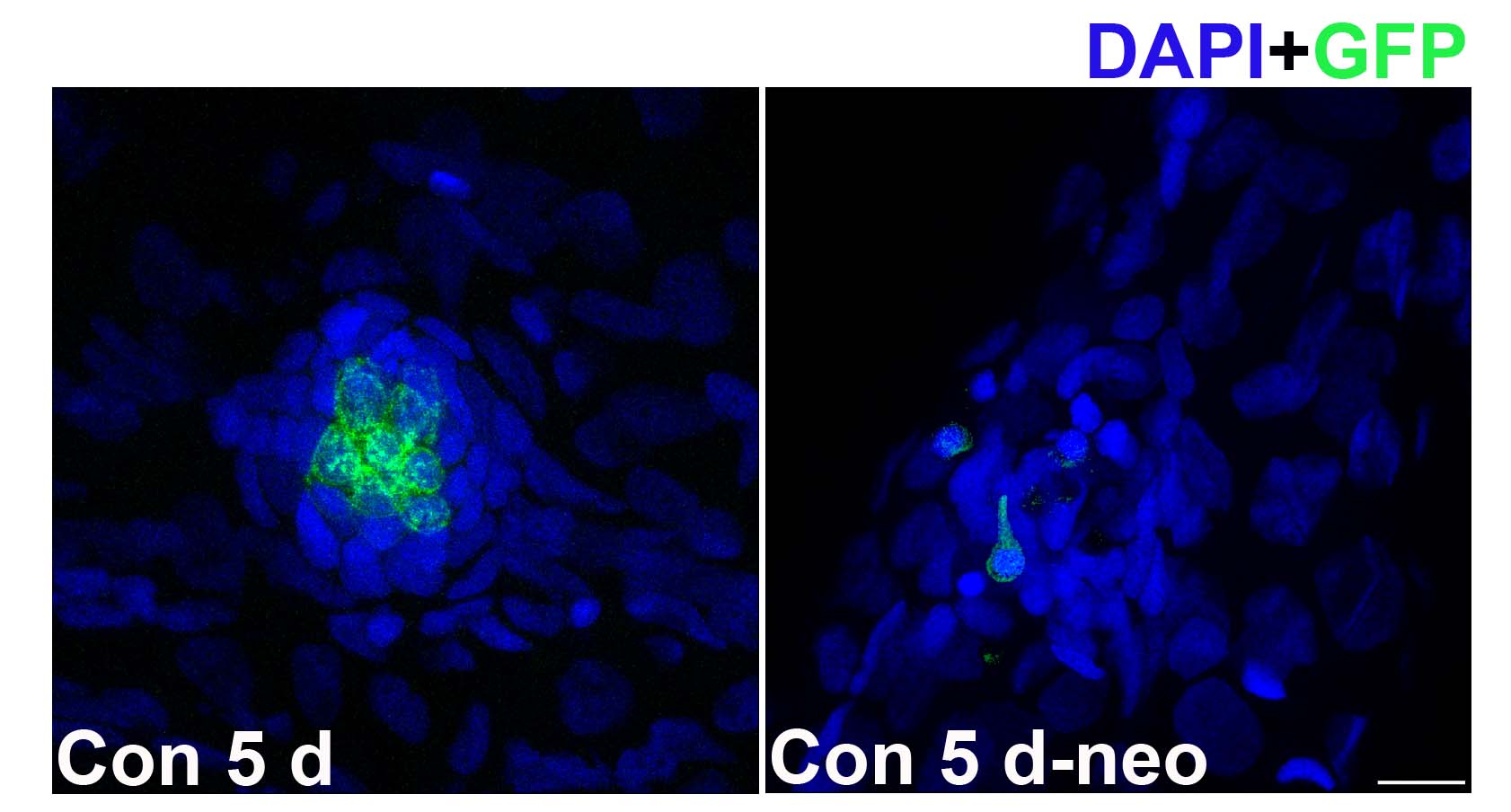

Supplement: Supplementary Figure 1 — Five dpf zebrafish larvae treated with 400 μM neomycin to kill mature lateral line hair cells. [file Image1.jpg]

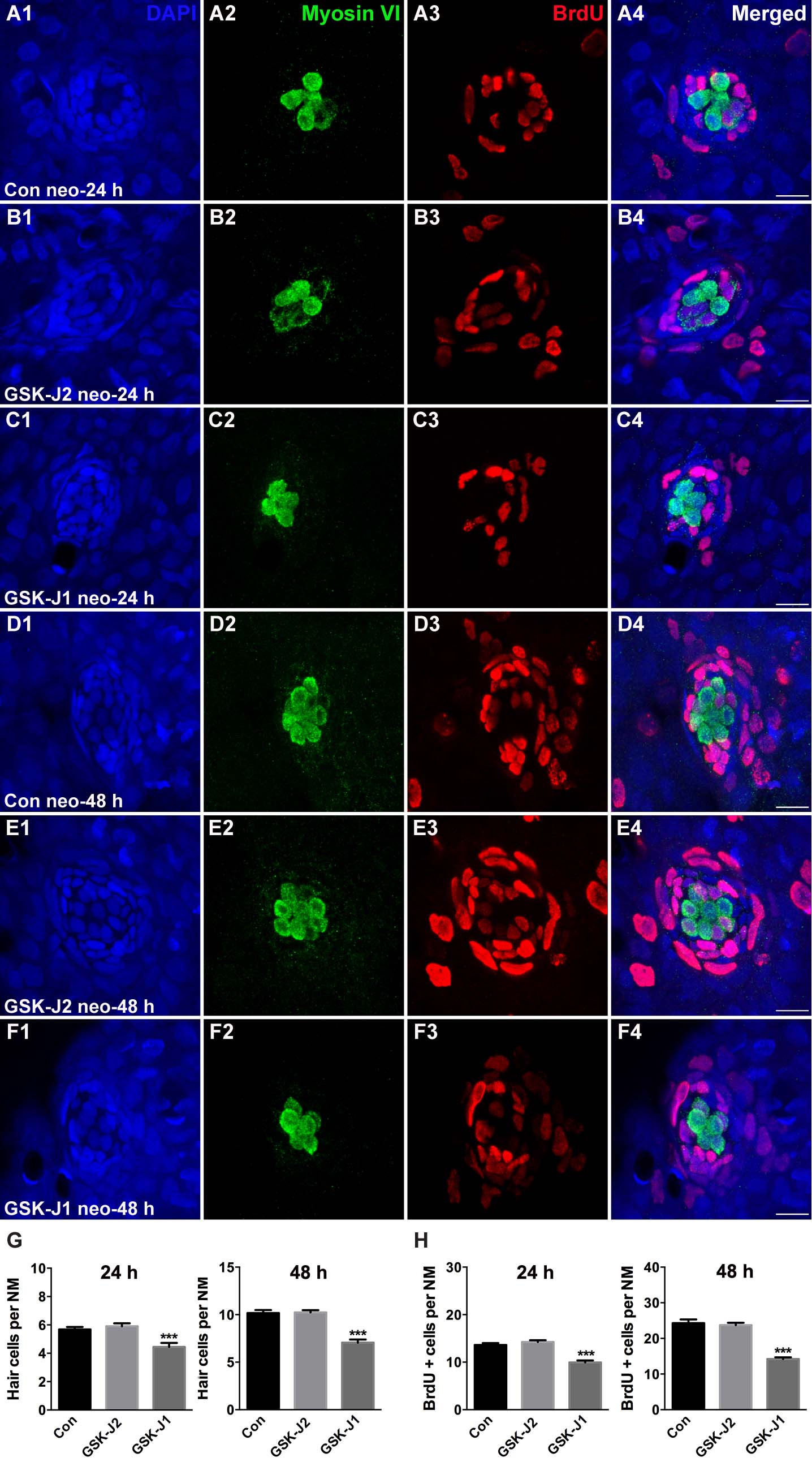

Supplement: Supplementary Figure 2 — GSK-J1 impaired zebrafish hair cell regeneration. (A–F) 5 dpf larvae were treated with 400 μM neomycin for 1 h followed by GSK-J1 exposure for 24 or 48 h in the presence of BrdU. GSK-J1 significantly reduced the numbers of myosinVI-positive (green) hair cells and BrdU-positive (red) replicating cells. Scale bars = 10 μm. (G,H) Quantification of myosinVI-positive and BrdU-positive cells per neuromast (NM) in DMSO-treated control larvae (Con), 15 μM GSK-J2-treated control larvae, and 15 μM GSK-J1-treated larvae at 24 or 48 h following neomycin damage. In the 24-h group, n = 28 neuromasts of DMSO-treated control larvae (14 larvae), n = 30 neuromasts of 15 μM GSK-J2-treated control larvae (15 larvae), and n = 20 neuromasts of 15 μM GSK-J1-treated larvae (10 larvae); in the 48-h group, n = 18 neuromasts of DMSO-treated control larvae (9 larvae), n = 22 neuromasts of GSK-J2-treated control larvae (11 larvae), and n = 18 neuromasts of 15 μM GSK-J1-treated larvae (9 larvae). ***p < 0.0001. Bars are mean ± sem. [file Image2.JPEG]

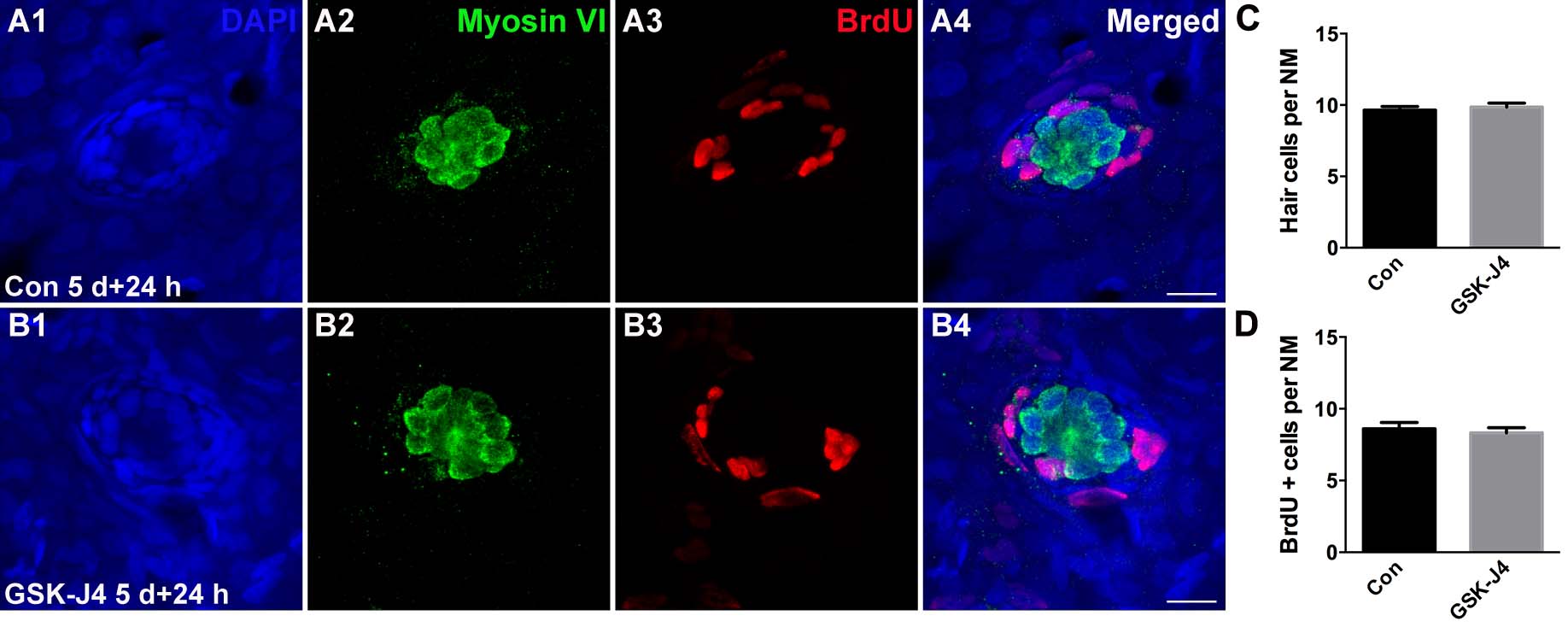

Supplement: Supplementary Figure 3 — GSK-J4 incubation did not affect the pattern of cell proliferation during development. (A,B) In the larvae not exposed to neomycin, supporting cell proliferation was at a low level overall for both DMSO-treated control and GSK-J4-treated larvae. (C,D) Quantification of myosinVI-positive hair cells and BrdU-positive cells per neuromast (NM) in DMSO-treated 5 dpf control larvae (Con) and 10 μM GSK-J4-treated 5 dpf larvae for 24 h. n = 20 neuromasts of DMSO vehicle control larvae (10 larvae) and n = 28 neuromasts of 10 μM GSK-J4-treated larvae (14 larvae). [file Image3.JPEG]

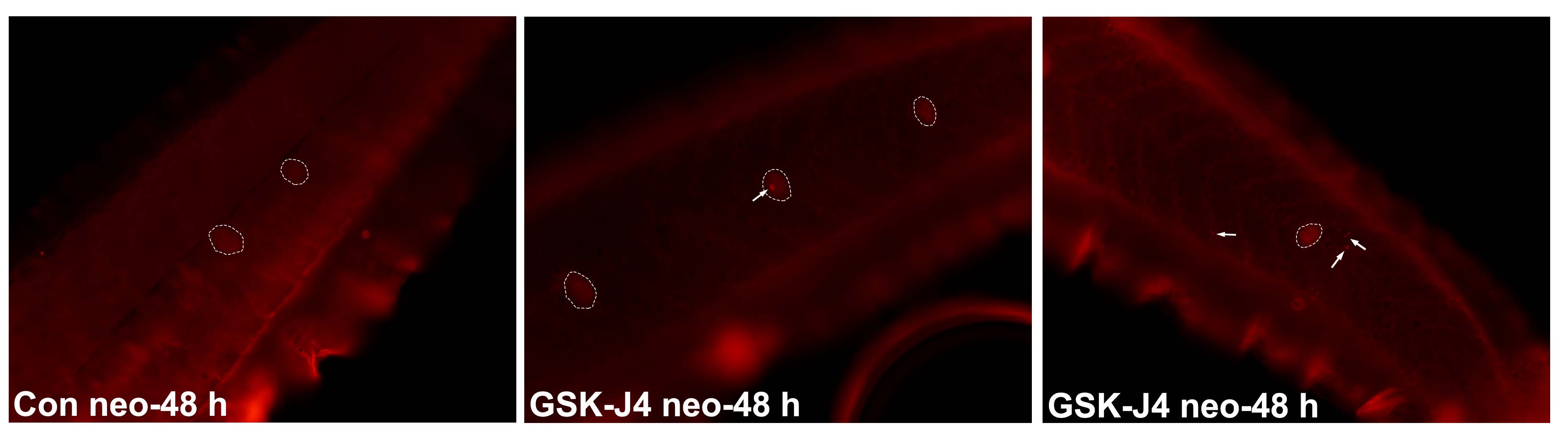

Supplement: Supplementary Figure 4 — Effects of GSK-J4 on apoptosis in the zebrafish body. Detection of cell apoptosis by cleaved caspase-3 staining in the body of zebrafish larvae exposed to DMSO (Con) or 10 μM GSK-J4 at 48 h following neomycin damage. The cleaved caspase-3-positive cells are indicated by white arrows, and the neuromasts are outlined. [file Image4.JPEG]

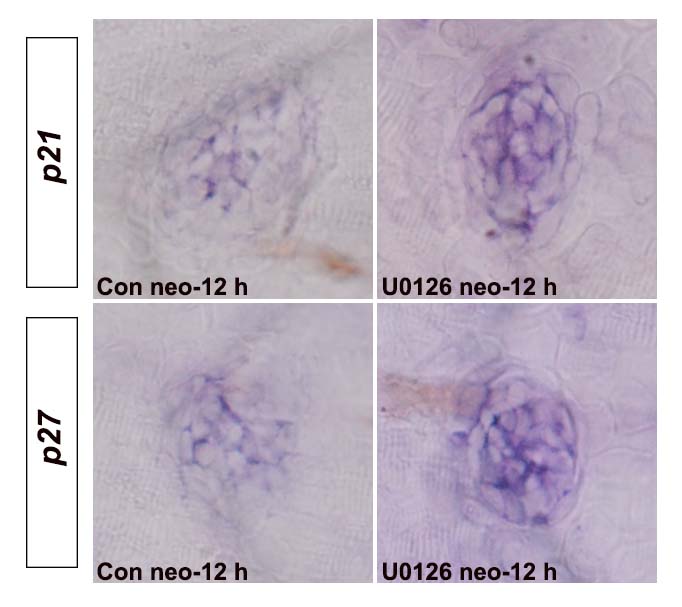

Supplement: Supplementary Figure 5 — Effects of ERK1/2 inhibition on the expression of p21 and p27. The mRNA levels of p21 and p27 in regenerating neuromasts were increased after U0126 treatment at 12 hpt when compared to the respective control larvae (n = 16–20 neuromasts per group). [file Image5.JPEG]
